# Untargeted Metabolomics Reveals Molecular Effects of Ketogenic Diet on Healthy and Tumor Xenograft Mouse Models

**DOI:** 10.3390/ijms20163873

**Published:** 2019-08-08

**Authors:** David Licha, Silvia Vidali, Sepideh Aminzadeh-Gohari, Oliver Alka, Leander Breitkreuz, Oliver Kohlbacher, Roland J. Reischl, René G. Feichtinger, Barbara Kofler, Christian G. Huber

**Affiliations:** 1 Bioanalytical Research Laboratories, Department of Biosciences and Cancer Cluster Salzburg, University of Salzburg, Hellbrunnerstraße 34, 5020 Salzburg, Austria; 2 Research Program for Receptor Biochemistry and Tumor Metabolism, Department of Pediatrics, Paracelsus Medical University, 5020 Salzburg, Austria; 3 Applied Bioinformatics, Department of Computer Science, University of Tübingen, 72076 Tübingen, Germany; 4 Institute for Bioinformatics and Medical Informatics, University of Tübingen, Sand 14, 72076 Tübingen, Germany; 5 Institute for Translational Bioinformatics, University Hospital Tübingen, 72076 Tübingen, Germany; 6 Biomolecular Interactions, Max Planck Institute for Developmental Biology, Max-Planck-Ring 5, 72076 Tübingen, Germany

**Keywords:** ketogenic diet, breast cancer, xenograft, untargeted metabolomics, HPLC-MS, reversed phase chromatography, hydrophilic liquid interaction chromatography

## Abstract

The application of ketogenic diet (KD) (high fat/low carbohydrate/adequate protein) as an auxiliary cancer therapy is a field of growing attention. KD provides sufficient energy supply for healthy cells, while possibly impairing energy production in highly glycolytic tumor cells. Moreover, KD regulates insulin and tumor related growth factors (like insulin growth factor-1, IGF-1). In order to provide molecular evidence for the proposed additional inhibition of tumor growth when combining chemotherapy with KD, we applied untargeted quantitative metabolome analysis on a spontaneous breast cancer xenograft mouse model, using MDA-MB-468 cells. Healthy mice and mice bearing breast cancer xenografts and receiving cyclophosphamide chemotherapy were compared after treatment with control diet and KD. Metabolomic profiling was performed on plasma samples, applying high-performance liquid chromatography coupled to tandem mass spectrometry. Statistical analysis revealed metabolic fingerprints comprising numerous significantly regulated features in the group of mice bearing breast cancer. This fingerprint disappeared after treatment with KD, resulting in recovery to the metabolic status observed in healthy mice receiving control diet. Moreover, amino acid metabolism as well as fatty acid transport were found to be affected by both the tumor and the applied KD. Our results provide clear evidence of a significant molecular effect of adjuvant KD in the context of tumor growth inhibition and suggest additional mechanisms of tumor suppression beyond the proposed constrain in energy supply of tumor cells.

## 1. Introduction

Since ketogenic diet (KD) was established in the 1920s to treat epilepsy [1], it has been under investigation for many years as a potential therapy for numerous other diseases, such as Parkinson’s disease, Alzheimer’s disease, acne, and diabetes [2,3,4,5,6]. KD is able to regulate hormones and growth factors. For example, KD reduces the levels of insulin and insulin-like growth factor (IGF-1) [7]. In the view of tumor formation and growth, the most important functions of the growth factors of the IGF family are that they enhance both cell proliferation and escape from apoptosis [8]. Furthermore, KD can stimulate the cellular endogenous anti-oxidant system by activating the nuclear factor erythroid-derived 2 (NF-E2)-related factor 2 (Nrf2), an important inducer of detoxification genes [9]. In the context of tumor therapy, KD has been propounded to potentiate antitumor effects of chemotherapy or radiation and to simultaneously reduce needed dosages to increase the quality of life of patients [10,11,12]. In the long tradition of applying this diet, various forms have been developed mainly differing in the fat:carbohydrate:protein ratio and in the type of fat used. A traditional KD consists of a 4:1 ratio (fat:carbohydrate + protein). An alternative to the traditional KD is, for example, a KD supplemented with medium-chain triglyceride (MCT). Compared to long-chain triglycerides (LCT), MCTs are more rapidly absorbed into the bloodstream and oxidized for energy delivery because of their ability to passively diffuse through membranes. Moreover, MCTs have higher potency to increase ketone body synthesis in the liver. Thus, adding MCTs to a KD might allow a less strict diet [13,14].

Cancer cells exhibiting the well-known “Warburg effect” are highly dependent on glucose as an energy source, and show frequently low but functional oxidative phosphorylation (OXPHOS). Even in the presence of oxygen, they manage their energy supply mainly via aerobic glycolysis, where pyruvate is oxidized to lactate instead of being fueled into the citric acid cycle [15]. Targeted metabolomics studies have already shown that KD leads to elevated levels of ketone bodies in plasma, derived from β-oxidation of fatty acids [16]. During fasting or under a KD, acetoacetate and β-hydroxybutyrate, the predominant products of ketogenesis, are supposed to replace glucose as the primary source of energy. After conversion to acetyl-CoA, they are introduced into the citric acid cycle, where NADH is generated to drive ATP production via oxidative phosphorylation, which occurs in the inner mitochondrial membrane. Regarding cancer, tumor cells are supposed to be strongly constricted in energy production by a combination of minimal carbohydrate and extensive fat uptake, while energy supply of normal cells is assured via ketone bodies [10,17].

Neuroblastoma represents a cancer type, for which the application of KD has already revealed promising results in terms of inhibition of tumor growth [18,19]. In contrast, for breast cancer so far there are not many preclinical studies available where KD was used [20,21,22]. A recent study indicated that KD enhances the efficacy of targeted therapy, in particular phosphatidylinositol-3 kinase (PI3K) inhibitors, and overcomes drug resistance [20]. In humans only a couple of case reports are available where KD was applied in combination with other treatments [23,24]. One study showed that short term KD during radiotherapy was beneficial (progression and quality of life) for a breast cancer patient [25]. Since several types of breast cancer are characterized by the Warburg effect, and the KD mimics a fasting diet, with the energy supply coming mostly through the catabolism of ketone bodies [26], we hypothesized that application of a MCT supplemented KD as an auxiliary treatment might also be beneficial in breast cancer therapy. Untargeted metabolomics (MTX) represents a powerful tool to study biological processes induced by specific stimuli on the metabolome of model organisms both in vitro and in vivo [27,28]. Due to its close association with the phenotype, metabolic profiling might provide the most confident fundament for the correlation of observations made on the level of phenotype with global changes in biomolecules and/or molecular events [29,30]. This is especially important in cases where little is known about the putative molecular effects of a certain treatment. Here, untargeted analysis of the metabolome can elucidate changes of canonical pathways, facilitating generation of hypotheses that can subsequently be verified via follow-up experiments [31]. 

Besides the induction of ketone body formation [10] and changes in amino acid metabolism [16], global effects of KD on the metabolome are widely unknown, particularly in relation to tumor growth. Therefore, we here implement xenografts of human tumors as cancer models to study the effect of KD in combination with classical chemotherapy. In order to cover the broadest possible range of metabolic characteristics, we examined biological effects of the therapy by untargeted HPLC-MS-based metabolomic analysis, employing four different and orthogonal combinations of chromatographic selectivity and mass spectrometric ionization. Using statistical data evaluation for the detection of significantly regulated metabolites, we aimed at discovering biological networks and pathways involved in the adaptation to KD in combination with chemotherapy. In that course, further factors induced by KD that lead to impaired tumor proliferation, beside the proposed induction of lowered glucose blood levels could be revealed.

## 2. Results and Discussion

### 2.1. Study Design

In order to investigate the effects of KD on tumor growth in combination with cytostatic therapy, the plasma metabolomes of a human xenograft breast cancer model in mice were analyzed. Although it would be interesting to investigate metabolites locally in tumor tissue, this has its limitations as tumor tissue is frequently heterogeneous, and the microenviroment consisting of tumor-associated macrophages (TAMs), tumor associated fibroblasts (TAFs), and the vascular system would also contribute to the data. Thus, such data might not only reflect changes in tumor cells per se. On the other hand, the usefulness of plasma/serum metabolomics for biomarker discovery in cancer research has been proven numerously [32]. In light of the currently established methodology, we believe that the conducted analysis of plasma samples represents the best choice for the demonstration of systemic alterations caused by adjuvant KD and can be thereby seen as a proof of principle.

**Figure 1:**
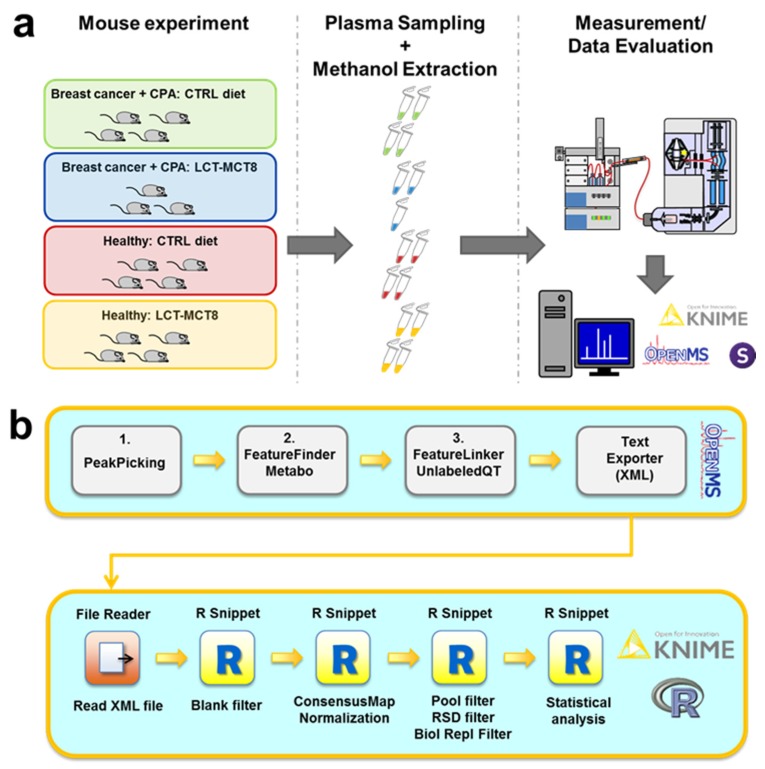
General (**a**) and computational workflow (**b**) applied for metabolomics studies. CPA—cyclophosphamide; LCT-MCT8—long chain triglyceride/medium-chain triglyceride diet. For details of the computational workflow, see the Materials and Method section.

The MTX samples were extracted from the plasma of anesthetized mice. All animals bearing tumor xenografts received cyclophosphamide (CPA) chemotherapy. Plasma of healthy vs. tumor bearing mice samples, which received either ketogenic or normal diets, was analyzed. As a previous study revealed that a combination of long-chain triglycerides (LCT) and medium-chain triglycerides (MCT8) exerted the most potent effect on inhibition of tumor growth [19], we decided to use a LCT-MCT8 diet in the treatment experiments. Healthy mice, receiving neither chemotherapy nor KD, served as negative controls (Figure 1a). 

In order to maximize the number of confident metabolite identifications, we employed chromatographic separation both by reversed-phase high-performance liquid chromatography (RP-HPLC) and hydrophilic interaction liquid chromatography (HILIC) in combination with detection by electrospray ionization mass spectrometry (ESI-MS) both in positive (posESI) or negative (negESI) detection mode, as previously described [27,31]. Very stringent filtering for elimination of background signals and unstable signals utilizing custom workflows (Figure 1b) based on open-source software was performed as previously explained [27]. After detection of significantly regulated metabolite features, metabolite identification was performed on the basis of fragmentation spectra with the open-source tools Metlin, SIRIUS, and CSI:FingerID [33,34,35,36,37]. Finally, standard compounds were analyzed by RP-HPLC/HILIC-pos/negESI-MS/MS, yielding a database of 46 different metabolites with corresponding retention times and mass spectra (see Supplementary Material), which were utilized for metabolite identification at level 1 according to the criteria defined by the Metabolomics Standards Initiative [38]. Accordingly, in this article the term “metabolite” is only used for signals that have been unambiguously identified via comparison with reference standards, while all others are referred to as “features”. Finally, biological pathway analysis using the KEGG database [39,40,41] was performed to reveal the metabolic effects of the different treatments.

### 2.2. Ketogenic Diet Induced Effects in MDA-MB-468 Tumor Bearing Mice

LCT-MCT8 diet (ratio of fat:carbohydrate + protein = 8:1; LCTs enriched with 25% 8-carbon MCTs; Supplementary Table S2) and cyclophosphamide (CPA) 30 mg/kg/day treatments significantly decreased tumor volume in MDA-MB-468 xenografts bearing mice already after the first 20 days of treatment, compared to the mice treated with CPA and control (CTRL) diet (Supplementary Figure S1a). KD was well tolerated, after an initial slight weight loss, due to adaptation to the different taste and palatability of the new diets, the mice body weight stabilized and remained quite constant throughout the experiment (Supplementary Figure S1b).

LCT-MCT8 diet significantly increased the concentration of blood ketone bodies in the mice already after five days of treatment (for breast cancer bearing mice see Supplementary Figure S1c; for healthy mice see Supplementary Figure S1d in [42]). In contrast, average blood glucose levels did not show any diet specific change and remained mostly unchanged until termination of the experiment after 80 days (Supplementary Figure S1d).

### 2.3. Results of Untargeted Metabolome Analysis

Combining features detected in all four measurement modes and strict filtering, we obtained approximately 7000 metabolite features in plasma of mice bearing breast cancer. This significant number of detected metabolite features represents a solid basis for further interpretation with regard to biological effects of the treatment of tumors by either chemotherapy or a combination of chemotherapy with KD. After recognition of significantly regulated features upon LIMMA (linear models for microarray data) [43] and Benjamini–Hochberg correction for multiple testing [44] (Table 1), the vast majority of detected features did not exert significant changes (corrected *p*-value < 0.05) in abundance comparing the different study groups. Subsequently, accurate masses of all differentially regulated metabolite features were compared with the human metabolome database (HMDB [45]) and putative molecular formulae were computed via SIRIUS, while the corresponding fragmentation spectra were interpreted using CSI:FingerID [33,34,35,36] and Metlin [37], which yielded 175 tentative metabolite identifications. After metabolite identification at level 1 using reference standards, a set of 32 identified and regulated metabolites was used for pathway analysis as described below (Supplementary Table S1).

### 2.4. Regulated Metabolites upon KD Treatment

**Figure 2:**
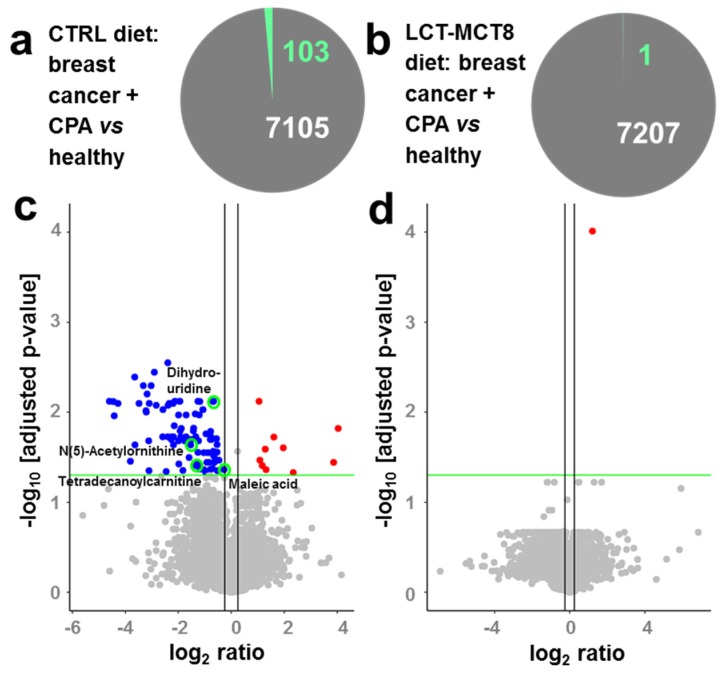
Pie charts (**a**,**b**) and corresponding volcano plots (**c**,**d**) showing significantly regulated features induced by breast cancer under chemotherapy in mice receiving a control diet (**a**,**c**) and a ketogenic diet (KD) (**b**,**d**), respectively. The volcano plots show the negative log *p*-value (linear models for microarray data (LIMMA) followed by Benjamini–Hochberg correction) against log_2_ ratios of regulations induced by breast cancer under chemotherapy. Blue dots represent down-regulated, red dots up-regulated, and grey dots non-regulated features with a significance threshold of 0.05 (green line). Vertical black lines indicate log_2_ ratios of −0.25 and 0.25. Green circles indicate identified metabolites.

**Figure 3:**
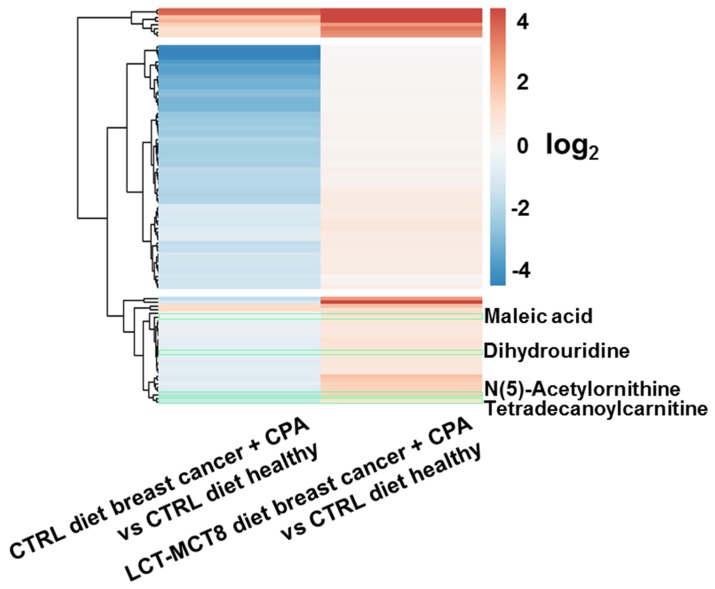
Heatmap of the 103 significantly regulated features induced by breast cancer under chemotherapy in mice receiving control or LCT-MCT8 diet. Green frames highlight the regulations of the four identified metabolites that are also highlighted in Figure 2c.

Comparing plasma of mice with breast cancer xenografts under chemotherapy with healthy mice, both receiving a control diet showed 103 significantly regulated features (Figure 2a), either induced by the tumor or the chemotherapy. This number decreased drastically when feeding a KD, revealing only one significantly regulated feature (Figure 2b). The observed decrease in regulated features suggests an approximation of the metabolism of mice bearing cancer and receiving chemotherapy to the metabolism of healthy mice, under an LCT-MCT8 diet. The volcano plots in Figure 2c,d clearly support this hypothesis, whereby the left plot represents a molecular fingerprint of the tumor under chemotherapy. Subsequently, when treated with LCT-MCT8 diet, all significant down-regulations induced by the breast cancer and/or chemotherapy in mice fed with a control diet disappeared, leaving only one significantly upregulated feature. Unfortunately, neither database search nor interpretation of fragment spectra allowed the assignment of a chemical structure of this feature having an accurate mass of 282.0442 Da. Nevertheless, the lack of significant regulations underlines the suppression of tumor and/or chemotherapy specific effects upon LCT-MCT8 diet. In this context, a heatmap (Figure 3) of the 103 significantly regulated features induced by the tumor and/or chemotherapy in mice fed with a control diet clearly confirms that the bulk of these regulations is reverted to the levels of healthy mice fed with a control diet or even inverted, when treated with an LCT-MCT8 diet. Figure 3 highlights the four identified metabolites maleic acid, 5,6-dihydrouridine, N(5)-acetylornithine, and tetradecanoylcarnitine, which were downregulated in the plasma samples of breast cancer bearing mice receiving the control diet but were slightly upregulated in the tumor model receiving KD (for more discussion see below).

Due to the fact that in breast cancer bearing mice under chemotherapy tumor size was reduced upon LCT-MCT8 diet, while glucose levels were not affected (Figure S1), our data suggests the induction of tumor growth inhibiting effects beyond the proposed constrain in energy supply of tumor cells. One limitation of this study is the fact that the mice used are not fully immunocompetent, as they preserve B-cells, neutrophils and macrophages, but they lack T-cells. Indeed, the immune system plays an important role in host defense against tumor cells. How the immune system is modulated by diets and metabolites is mostly unclear. However, Ni et al. have shown that a KD can correct the Th17/Treg imbalance of patients with childhood intractable epilepsy [46]. Thus, we speculate that KD by influencing immune cells might have an impact on tumor response. There is evidence that KD is able to increase the innate and adaptive immune responses against tumor cells, including cytolysis via tumor-reactive CD8+ T cells [47]. Furthermore, Formin et al. have shown that KD influences the mammalian target of rapamycin (mTOR) activity. mTOR regulates the innate immune function [48,49]. Thus, we hypothesize that the effect of KD on immunocompetent individuals will enhance the anticancer activity mediated by T-cells. Interestingly, using a brain tumor model, Zhou et al. have demonstrated KD to cause suppression of tumor growth in both immune active and immune deficient mice [17]. No major differences were present between the two strains: C57BL/6J and BALBc/J-SCI. For example, the influence of KD on body weight was equal in both strains. Blood glucose and β-hydroxybutyrate levels of the two strains showed a similar response to the KD, albeit the levels per se showed minor differences. Important to notice is that Zhou et al. did not use the same strain with or without immune system. To analyze the influence of the immune system only, isogenic mouse strains would be needed. 

**Figure 4:**
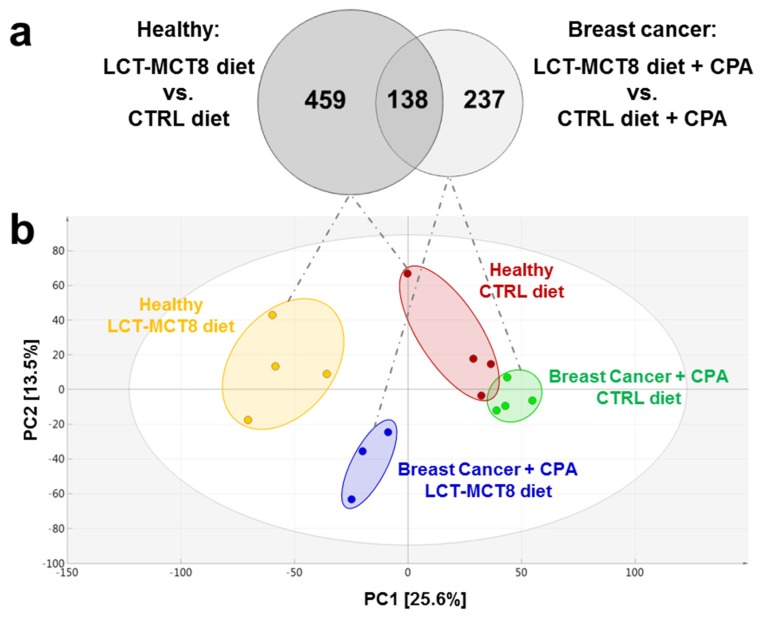
Overlap of significantly regulated features induced by LCT-MCT8 diet in breast cancer group vs. healthy group (**a**) and Principal Component Analysis (PCA) Score Scatter plots of plasma samples of breast cancer bearing mice (**b**).

Comparison of the mice bearing breast cancer xenografts under CPA treatment and receiving LCT-MCT8 diet with the ones fed with control diet revealed 375 significantly regulated features (Figure 4a, right circle of the Venn diagram). In healthy mice, the LCT-MCT8 diet induced 597 significant alterations (Figure 4a, left circle of the Venn diagram). The overlap between the two sets of regulated metabolite features was only 16.5% (138 features), whereas the bulk of regulated features differed between the two groups. This either indicates different effects of KD between healthy mice and those with breast cancer or different effects of KD in combination with chemotherapy.

Principal Component Analysis (PCA) of all detected features from all four modes of analysis (Figure 4b) unveiled distinct clustering of all four treatment groups included in the study, indicating that untargeted metabolomics represents a highly useful tool to visualize changes in the metabolome of biological model organisms upon certain treatments. The groups treated with KD clustered in the negative sector of PC1. The cohort of mice bearing breast cancer xenografts and receiving chemotherapy as well as control diet was found in the very right sector of the PCA. However, when treated with an adjuvant LCT-MCT8 diet this cohort shifted horizontally in between the group of healthy mice fed with a control diet and LCT-MCT8 diet, respectively. These observations reveal significant differences between the metabolomes of the treatment groups, which were finally confirmed by LIMMA followed by Benjamini–Hochberg correction to find regulated features to be subsequently identified via database searches and reference standards.

### 2.5. Regulations in Amino Acid Biosynthesis and N-Acetylation

**Figure 5:**
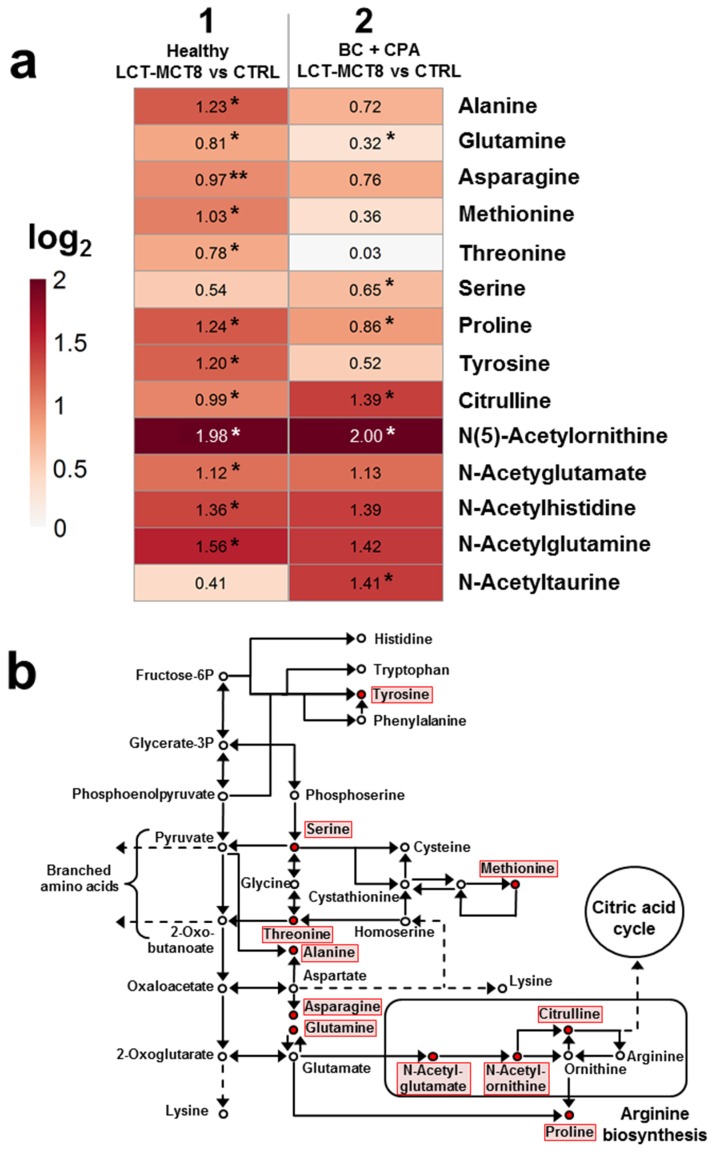
Heatmap of significantly regulated metabolites involved in amino acid biosynthesis induced by breast cancer under chemotherapy and KD, respectively (**a**) and pathway map of amino acid biosynthesis (**b**). Red dots in the pathway map [39,40,41] indicate metabolites showing a significant regulation induced by LCT-MCT8 diet in healthy and/or in breast cancer bearing mice.

Several metabolites involved in the amino acid biosynthesis pathway showed a significant up-regulation (LIMMA *p*-value after Benjamini–Hochberg correction < 0.05) upon treatment with LCT-MCT8 diet. The heatmap presented in Figure 5a illustrates the regulations of nine amino acids as well as five acetylated amino acids. The application of LCT-MCT8 diet generally led to an upregulation of the 14 metabolites in healthy mice (column 1 in Figure 5a). Moreover, in breast cancer bearing mice, the diet led to a rather moderate increase of most levels of the nine amino acids, while upregulation of N-acetylated amino acids was significantly more pronounced (column 2), which indicates a prominent role of acetylation in the context of KD in tumor bearing mice (see below).

The metabolic context of the altered amino acids is shown in Figure 5b. Since the observed increases (highlighted in red) concern a number of important nodes in the pathway, we conclude that increased amino acid biosynthesis represents one of the organism’s primary responses to a very limited supply of protein provided by the KD (8% in the LCT-MCT8 diet). However, a preclinical study on neuroblastoma does not support this hypothesis. No changes in the levels of specific AAs in the plasma of mice fed a control diet matching the low protein content of the KDs were observed [19]. Therefore, it seems that altered amino acid levels in the KD group are caused by cross-talk between fat- and protein-metabolic pathways. 

Another detail revealed in the metabolite regulations is the upregulation of only three of five important metabolites of the arginine biosynthesis, namely N-acetylglutamate, N(5)-acetylornithine, and citrulline. This suggests that high amino acid levels are not metabolized in the urea cycle, but are transferred to the citric acid cycle for further energy production. N-acetylation is mediated via N-acetyltransferases, which are important enzymes in the detoxification of medications and environmental toxins [50]. Due to the elevated levels of acetylated amino acids we assume that an increased expression or activation of N-acetyltransferases is induced by KD.

### 2.6. Regulation of N(5)-Acetylornithine and 5,6-Dihydrouridine

**Figure 6:**
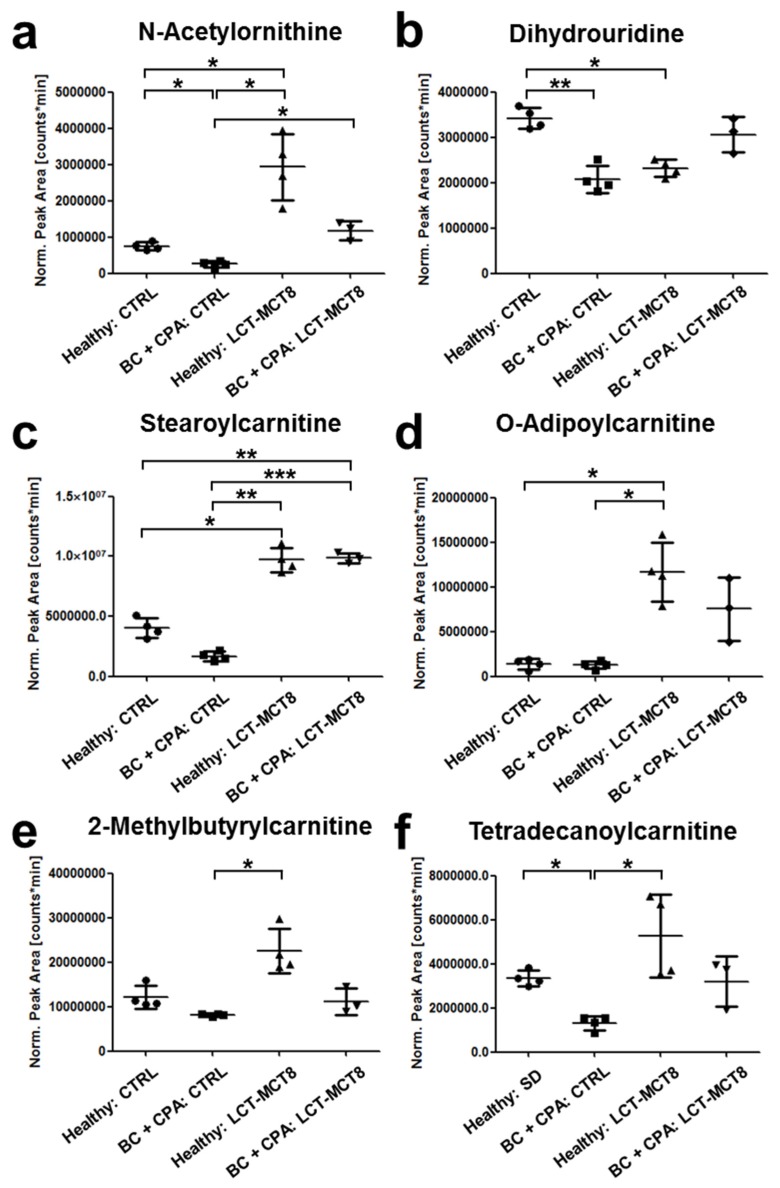
Regulation of several metabolites induced by KD and breast cancer xenografts. Graphs show normalized peak areas of N(5)-acetylornithine (**a**), 5,6-dihydrouridine (**b**), stearoylcarnitine (**c**), O-adipoylcarnitine (**d**), 2-methylbutyrylcarnitine (**e**) and tetradecanoylcarnitine (**f**).

Of all the metabolites shown in the heatmap of Figure 5a, particular interest applies to N(5)-acetylornithine, which was one of the four identified metabolites showing a significant regulation comparing breast cancer bearing mice and healthy mice, both receiving a control diet (log_2_ ratio = −1.54; *p*-value LIMMA after Benjamini–Hochberg correction = 0.024, Figure 6a). Compared to healthy/control diet, N(5)-acetylornithine is significantly down-regulated in the CPA-treated breast cancer mice (Figure 6a). KD inverts this into significant up-regulation, albeit total levels remain lower than in healthy, LCT-MCT8 treated mice. This implies that the down-regulation of N(5)-acetylornithine was induced by the breast cancer xenograft, independent of the applied diet. Moreover, the extent of up-regulation induced by the LCT-MCT8 diet is considerably higher as compared to the regulation of the residual metabolites involved in amino acid biosynthesis. As can be seen in Figure 6a, the decrease of N(5)-acetylornithine levels caused by the tumor under chemotherapy is counter-balanced by the LCT-MCT8 diet, so that the N(5)-acetylornithine concentration is restored to levels similar to those in healthy mice fed with a control diet.

N(5)-acetylornithine is a minor component of deproteinized mammalian plasma and known to be an intermediate in the arginine biosynthesis and in the urea cycle as a precursor of ornithine (Figure 5b) [51]. Recently, increased concentrations of N(5)-acetylornithine in urine of mice have been associated with aging effects. In that study, the authors suggested a correlation with a malfunction of the urea cycle and subsequent diminished removal of ammonia from the body [52]. In this context, Suhre et al. showed a connection of N-acetylated ornithine with kidney function, whereby elevated concentrations were associated with lower estimated glomerular filtration rates [50]. McClay et al. found N(5)-acetylornithine to be down-regulated by treatment of mice with haloperidol, an antipsychotic drug [53]. In contrast, Napoli et al. observed significantly higher N(5)-acetylornithine levels in human plasma of X-associated tremor/ataxia syndrome (FXTAS) patients after treatment with allopregnanolone, a natural neurosteroid that induces beneficial effects in neurodegenerative diseases [54]. However, none of the studies were able to provide a causal biological interpretation for the observed alterations. The few published data suggest that elevated N(5)-acetylornithine concentrations predominantly characterize diseased conditions. This is in contrast to our observation, where cancer seems to reduce N(5)-acetylornithine levels, which can be brought back to normal levels with the help of KD.

The 5,6-dihydrouridine concentration is significantly lower in plasma of mice bearing breast cancer and receiving chemotherapy compared to healthy mice (Figure 6b). 5,6-Dihydrouridinylation is a posttranscriptional modification of tRNA molecules, providing the required conformational flexibility to enable loop formation and tertiary interactions at the same time [55]. Upon metabolomic serum analysis, elevated 5,6-dihydrouridine has been suggested as a potential diagnostic marker for squamous cell carcinoma [56], which contrasts our results of reduced concentration. Moreover, in mice bearing breast cancer xenografts and receiving an adjuvant LCT-MCT8 diet, levels of 5,6-dihydrouridine were restored to the range of healthy mice receiving control diet, which again corroborated the effect of KD to support homeostasis. Interestingly, in healthy mice LCT-MCT8 diet also caused decreased concentrations of 5,6-dihydrouridine compared to the control diet.

### 2.7. Fatty Acid Transport

Carnitine is known as a key metabolite relevant for the transport of fatty acids into mitochondria, where their degradation via ß-oxidation takes place. Thereby, the fatty acid chain is attached to carnitine via an ester bond, resulting in an acylcarnitine [57,58]. As can be seen in Figure 6c–f, several acylcarnitine derivatives were significantly regulated by KD. Along that line, stearoylcarnitine O-adipoylcarnitine and 2-methylbutyrylcarnitine levels were elevated by the LCT-MCT8 diet (Figure 6c–e). Due to the high dietary fat uptake during a KD, increased transport and metabolism of fatty acids are reasonable and in accordance with the literature [59]. Since such changes must be expected in diets with high fat content, this molecule rather poses an example for the meaningfulness of data generated by our metabolomics workflow.

On the other hand, tetradecanoylcarnitine showed significant downregulation in the breast cancer-bearing cohort fed with control diet (Figure 6f), revealing that the fatty acid metabolism is partially dysregulated by the tumor and/or chemotherapy. This trend was also observed for stearoylcarnitine, which is in accordance with the publication of Wu et al., who found decreased levels of stearoylcarnitine and other metabolites involved in fatty acid metabolism in a lung cancer mouse model [57]. In this respect, our data suggests that the downregulation of tetradecanoylcarnitine and stearoylcarnitine induced by the tumor under chemotherapy is balanced or even reversed by KD, since both metabolites trend towards increased concentrations in healthy as well as in breast cancer bearing mice when treated with chemotherapy and an LCT-MCT8 diet.

## 3. Materials and Methods

### 3.1. Cell Culture

MDA-MB-468 cell line (ATCC, HTB-132) was used for the generation of breast cancer xenografts. Cells were cultivated in high glucose DMEM medium (Sigma-Aldrich, St. Louis, MO, USA) supplemented with heat-inactivated fetal bovine serum (Gibco, Vienna, Austria) and penicillin/streptomycin amphotericin B solution (Lonza, Cologne, Germany).

### 3.2. Animal Experiments

All in vivo experiments were performed in accordance with protocols approved for this study by the Salzburg Animal Care and Use Committee (20901-TVG/116/6-2016, approved on 19 September, 2016 for breast cancer and 20901-TVG/87/7-2014, approved on 28 July, 2014 for healthy animals). Mice were maintained under specific pathogen-free conditions and care conformed to the Austrian Act on Animal Experimentation. All experiments were performed on female CD-1 nu/nu mice (Charles River, Sulzfeld, Germany), the animals were group-housed and had unlimited access to food and water. Xenografts were established as previously described [19,42]. Briefly, a suspension of 1.5 × 10^7^ MDA-MB-468 cells in serum-free medium and matrigel (BD Biosciences, Austria) were injected into the right flank of 5- to 6-week-old mice. 

As soon as tumor size reached 300–350 mm^3^ (i.e., 2–5 weeks after injection of tumor cells), mice were randomized into different dietary intervention groups (CTRL, LCT-MCT8; *n* = 5–6) [19]. As mice are able to keep blood glucose levels and show lower ketosis on 2:1 to 4:1 diets, compared to humans, we decided to use an 8:1 diet in our mouse model, to reach at least a ketosis over 2 mmol/L. Dietary interventions were combined with oral metronomic chemotherapy with CPA (30 mg/kg). The healthy, 7 weeks old, mice were fed with the experimental diets (CTRL, LCT-MCT8; Supplementary Table S2) [19,42]. All animals were monitored twice a week for body weight using a digital scale. Blood glucose and ketone body (β-hydroxybutyrate) levels were monitored once a week using a specific enzyme-based kit (Precision Xceed, Abbott Laboratories, Vienna, Austria). Tumor volume was measured twice a week in the xenografts bearing mice, by using a caliper and calculating the volume according to the formula 4/3 Pi × d_1_/2 × d_2_/2 × d_3_/2 (d—dimension).

Breast cancer bearing mice were euthanized after 80 days of treatment, whereas healthy mice after 40 days. Therefore, mice were injected with 10 μL/g of anesthetic mix (ketamine 20.5 mg/mL, xylazine 5.4 mg/mL, acepromazine 270 μg/mL in saline solution), and after checking for absence of reflexes from the paw, heart puncture was performed. Mice were then immediately euthanized via head-neck dislocation. Blood was transferred into tubes (BD Microtainer^®^ PSTTM LH tubes; BD Biosciences, Vienna, Austria) and plasma was collected as described in the manufacture’s protocol, and snap frozen in liquid nitrogen [19,42].

### 3.3. Preparation of Plasma Samples

Metabolites were extracted by adding 810 µL of ice-cold methanol (VWR, Rednor, Pennsylvania, USA), containing 10 µmol L^−1^ ethylparaben (Fluka, Buchs, Switzerland), 2 µmol L^−1^ 3-nitro-L-tyrosine (Sigma-Aldrich, St. Louis, MO, USA), and 4 µmol L^−1^ d_4_- succinate (Sigma-Aldrich) to 90 µL of plasma. The internal standards ethylparaben, 3-nitro-L-tyrosine and d_4_-succinate were used for quality control for the assessment of the system stability. Proteins were pelleted in a centrifuge (HERMLE, Wehingen, Germany) at 18,620 rpm for 10 min at 4 °C. The supernatant was evaporated to dryness in a vacuum concentrator (Eppendorf, Hamburg, Germany) at room temperature, followed by resuspension in 90 µL 50% methanol.

### 3.4. HPLC-MS Measurements

An ultra-high-performance liquid chromatography (UHPLC) system consisting of an Accela 1250 pump, a Column Oven 300 (all from Thermo Fisher Scientific, Bremen, Germany), and an LC PAL DLW Option Autosampler with a 100 µL syringe (from CTC Analytics AG, Zwingen, Switzerland) was coupled to a hybrid quadrupole-Orbitrap mass spectrometer (Model Q Exactive Plus; Thermo Fisher Scientific) equipped with a heated electrospray ion source operating in the positive or negative ion mode. A quality control sample (pool) was generated by merging an aliquot (10 µL) of all samples included in the study. Prior to injection into the HPLC-MS system, samples and pools were diluted 1:5 with Millipore water for reversed phase mode and 1:3 with acetonitrile (VWR, Rednor, PA, USA) for HILIC mode separation.

Each sequence started with three blank runs, followed by three pool injections and finished with one pool and one blank run. In between, a blank run and a pool run were conducted after every third sample. Each sample was measured in four different selectivity and ionization mode combinations, which were RP—positive ionization, RP—negative ionization, HILIC—positive ionization, and HILIC—negative ionization, resulting in four data sets per study. For each combination an exclusion list of the 100 most abundant ions obtained from prior blank runs was generated, to avoid MS fragmentation of chemical or electronic noise signals.

For RP-HPLC separations, a 100 × 2.1 mm i.d. Hypersil Gold aQ column (Thermo Fisher Scientific) packed with 1.9 µm octadecyl silica particles was applied. For column protection, a 4.0 × 3.0 mm i.d. C_18_ Security Guard pre-column (Phenomenex, Torrance, CA, USA) was installed. In reversed phase mode, mobile phase A and B were Millipore water and acetonitrile, both containing 0.10% formic acid (Sigma-Aldrich). The HPLC method started with holding 100% A for 1.5 min, followed by a linear gradient to 100% B in 6.5 min. After washing for 2.0 min at 100% B, the column was re-equilibrated to starting conditions for 3.0 min, resulting in a total run time of 13.0 min.

HILIC-HPLC separations were performed using a 150 × 2.0 mm i.d. Nucleodur HILIC column (Macherey-Nagel, Düren, Germany), packed with 1.8 µm zwitterionic functionalized particles. Additionally, a 4.0 × 2.0 mm i.d. Nucleodur HILIC 1.8 µm pre-column, also from Macherey-Nagel was applied to protect the column. In the HILIC mode, mobile phase A contained 50 mM ammonium formate in 50% acetonitrile, while mobile phase B was 10 mM ammonium formate (Sigma-Aldrich) in 90% acetonitrile. After holding 100% B for 3.0 min a linear gradient to 100% A in 17.0 min was applied. The column was washed for 2.0 min at 100% A and re-equilibrated to starting conditions for 8.0 min, resulting in a total run time of 30.0 min.

In both modes a flow rate of 0.30 µL min^−1^ and an injection volume of 2.70 µL were applied. The column temperature was held constant at 30 °C. Apart from the run time of 13.0 min in RP mode and 30.0 min in HILIC mode, MS settings of the data-dependent Top 5 method were identical for both selectivity modes. Scan range and resolution of MS^1^ scans were set to 80–850 *m*/*z* and 70,000, respectively, using an AGC target of 1 × 10^6^ and a maximum injection time of 50 ms. The five most abundant ions of the MS^1^ scan were isolated with an isolation window of 0.8 *m*/*z*, fragmented via HCD applying stepped normalized collision energies of 20, 40, 60 and acquired in centroid mode. The maximum injection time was set to 64 ms with an AGC target of 5 × 10^4^ at a resolution of 17,500. In addition to an exclusion of charges greater than two, dynamic exclusion of 5.0 s was used, in order to avoid multiple fragmentations of the same ions. Using these settings, all samples were measured in the positive and negative ionization mode in separated sequences for each selectivity and ionization mode. Tune parameters were a capillary temperature of 320 °C, a probe heater temperature of 350 °C and an S-lens level of 50 for both ionization modes. Differing settings were a sprayer voltage of 4.0 and 3.5 kV, sheath gas flow rate of 35 and 45, as well as an auxiliary gas flow rate of 5 and 10 in positive and negative ionization mode, respectively.

### 3.5. Data Evaluation

Acquired raw files were converted to mzML files using the MSConvert tool of the ProteoWizard software (version 3.0.8688). Metabolomics data have been deposited to the EMBL-EBI MetaboLights database [60] with the identifier MTBLS1066. The complete dataset can be accessed here https://www.ebi.ac.uk/metabolights/MTBLS1066. The resulting files were further processed using a bioinformatic workflow in the Konstanz Information Miner (KNIME) [61], version 3.3.4 with integrated OpenMS 2.1.0 software [33] (see Supplementary Tables S3–S7 for detailed settings). The applied KNIME workflow can be accessed here https://www.myexperiment.org/workflows/5109.html. After peak picking, feature detection and feature alignment, data was filtered, normalized, and statistically evaluated using Linear Models for Microarray Data (LIMMA) [43] followed by Benjamini–Hochberg correction [44] for multiple testing. 

The MTX study design and data treatment involved stringent filtering of “noise” signals or such originating from the chemical background, which enabled the reduction of chemical and electronic artefacts. Along that line, the HPLC-MS signals were aligned retention time wise, followed by exclusion of features that did not exceed a threshold of fivefold the median intensity of the corresponding signal in the blank from further data processing. In the next step, data was normalized via a robust regression, as explained in [27] to account for variations occurring during sample preparation and measurement. Features showing instable signals in the quality control runs and those not being present in a sufficient number of replicates or treatment conditions were removed. Filtering was based on peak area threshold in blank runs, as well as on relative standard deviation in quality control samples and occurrence in biological replicates.

Identification was done in a multiple step approach: (1) Monoisotopic mass search in the HMDB [45]; (2) molecular structure database search using SIRIUS in combination with CSI:FingerID [33,34,35,36]; (3) manual fragment spectra search using METLIN for all signals that resulted in a hit after step 1, but not after step 2 [37]; (4) verification of database hits via comparison of retention time and fragment spectra of reference standards. For detailed settings of the applied software used for identification see Supplementary Tables S8 and S9. Principal component analyses were generated using SIMCA (version 13.0.3.0; Umetrics, Umea, Sweden).

### 3.6. Verification of Database Hits with Standards

All reference standards, besides N(5)-acetylornithine, 5,6-dihydrouridine, and N-acetyltaurine were purchased from Sigma Aldrich and measured on the same instrument using the same methods as for plasma and tumor tissue samples. N(5)-acetylornithine and 5,6-dihydrouridine were customly synthesized by MedChemTronica (Sollentuna, Sweden) and AKos GmbH (Lörrach, Germany) respectively, while N-acetytaurine was synthesized inhouse. Retention time and MS^2^ spectra concordance between samples and standards was assessed manually by visual comparison.

## 4. Conclusions

The present study revealed molecular effects of KDs in a breast cancer mouse model. We could show that LCT-MCT8 diet can shift a derailed plasma metabolome induced by breast cancer xenografts under chemotherapy back to the one of healthy mice. Further, we found a strong up-regulation of amino acid metabolism as well as of amino acid N-acetylation in healthy and breast cancer bearing mice upon KD treatment. In this course, N(5)-acetylornithine, which is involved in amino acid biosynthesis as a precursor of arginine and proline was significantly down-regulated in plasma of the breast cancer xenograft group. LCT-MCT8 diet, however, induced an elevation of N(5)-acetylornithine levels back to the ones found in healthy mice fed with a control diet. This raises the interest for this metabolite and therefore requires further investigation to disclose its relevance in relation to tumor growth and treatment with adjuvant KD.

## Figures and Tables

**Table 1:** Number of significantly regulated metabolite features between differently treated mouse models.

	Number of Significantly Regulated Features		
Treatment Group	BC ^a^ + CPA ^b^ + LCT-MCT8 ^c^	Healthy + CTRL ^d^	Healthy + LCT-MCT8
BC + CPA + CTRL	375	103	1340
BC + CPA + LCT-MCT8	-	371	1
Healthy + CTRL	-	-	597

^a^ Breast cancer; ^b^ cyclophosphamide; ^c^ long-chain triglycerides/medium-chain triglyceride diet; ^d^ control diet.

## Supplementary Materials

Supplementary materials can be found at https://www.mdpi.com/1422-0067/20/16/3873/s1.

## Abbreviations

KD	ketogenic diet
IGF-1	insulin growth factor-1
NF-E2	nuclear factor erythroid-derived 2
Nrf2	nuclear factor erythroid-derived 2-related factor 2
OXPHOS	oxidative phosphorylation
PI3K	phosphatidylinositol-3 kinase
MTX	metabolomics
TAMs	tumor-associated macrophages
TAFs	tumor-associated fibroblasts
CPA	cyclophosphamide
CTRL	control
MCT8	medium-chain triglycerides
LCT	long-chain triglycerides
RP-HPLC	reversed-phase high-performance liquid chromatography
HILIC	hydrophilic interaction liquid chromatography
ESI-MS	electrospray ionization mass spectrometry
posESI	positive electrospray ionization
negESI	negative electrospray ionization
LIMMA	linear models for microarray data
HMDB	human metabolome database
mTOR	mammalian target of rapamycin
PCA	Principal Component Analysis
FXTAS	X-associated tremor/ataxia syndrome
UHPLC	ultra-high-performance liquid chromatography
KNIME	Konstanz Information Miner
