# Supplementary material for: Untargeted Metabolomics Reveals Molecular Effects of Ketogenic Diet on Healthy and Tumor Xenograft Mouse Models
Source: Int J Mol Sci. 2019 Aug 8;20(16):3873. doi: 10.3390/ijms20163873 (PMC6719192; doi:10.3390/ijms20163873)
Supplement: Supplementary file 1 [file ijms-20-03873-s001.zip › ijms-546015-supplementary-XML/ijms-546015-Supplementary table and figure.docx]

**Supplementary Information**

Untargeted Metabolomics Reveals Molecular Effects of Ketogenic Diet on Healthy and Tumor Xenograft Mouse Models

David Licha ^1^, Silvia Vidali ^2^, Sepideh Aminzadeh-Gohari ^2^, Oliver Alka ^3^, Leander Breitkreuz ^1^, Oliver Kohlbacher ^3,4,5,6^, Roland J. Reischl ^1^, René G. Feichtinger ^2^, Barbara Kofler ^2,^* and Christian G. Huber ^1,^*

^1^ Bioanalytical Research Laboratories, Department of Biosciences and Cancer Cluster Salzburg, University of Salzburg, Hellbrunnerstraße 34, 5020 Salzburg, Austria

^2^ Research Program for Receptor Biochemistry and Tumor Metabolism, Department of Pediatrics, Paracelsus Medical University, 5020 Salzburg, Austria

^3^ Applied Bioinformatics, Department of Computer Science, University of Tübingen, 72076 Tübingen, Germany

^4^ Institute for Bioinformatics and Medical Informatics, University of Tübingen, Sand 14, 72076 Tübingen, Germany

^5^ Institute for Translational Bioinformatics, University Hospital Tübingen, 72076 Tübingen, Germany

^6^ Biomolecular Interactions, Max Planck Institute for Developmental Biology, Max-Planck-Ring 5, 72076 Tübingen, Germany

* Correspondence: b.kofler@salk.at (B.K.); c.huber@sbg.ac.at (C.G.H.); Tel: +43 (0)5 7255-26274 (B.K.); +43 662 8044 5738 (C.G.H.); Fax: +43 (0)5 7255-58507 (B.K.); +43 662 8044 5751 (C.G.H.)


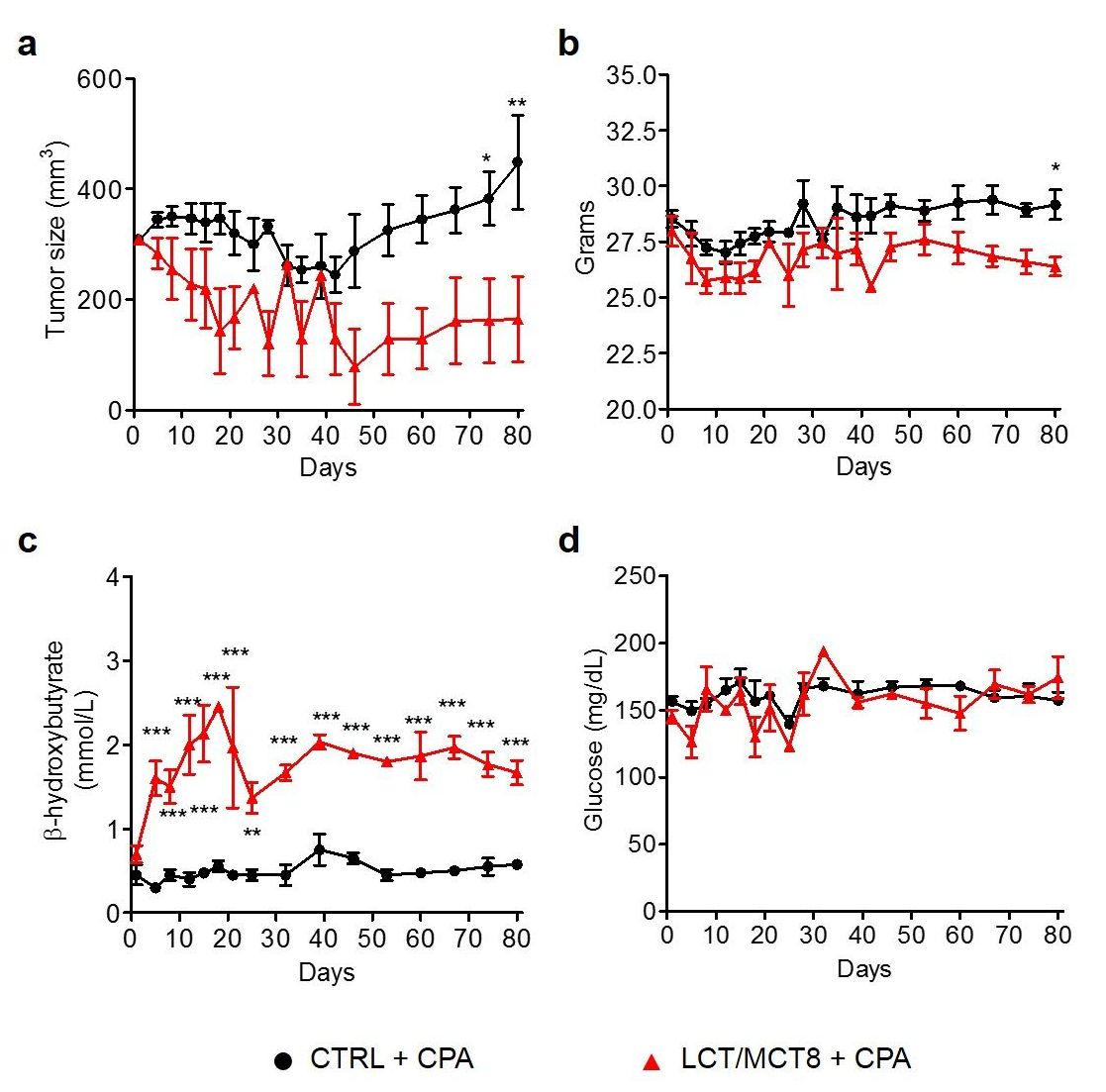


**Supplementary Figure S1.** Mean of the tumor size in MDA-MB-468 bearing mice under CTRL diet or LCT/MCT8 ketogenic diet (**a**). Average body weight variations in mice during dietary intervention (**b**). Average ketone bodies (mmol/L) (**c**) and blood glucose (mg/dL) levels (**d**) measured in blood of mice fed with the different diets. Data are given as mean ± SEM. Statistical analysis was performed by using two-way ANOVA (Dunnett's multiple comparison test), * *p* < 0.05, ** *p* < 0.01, *** *p* < 0.001; n = 5-6 mice.

**Supplementary Table S1.** Unambiguously identified metabolites *via* comparison with reference standards.

| **Molecule** | **Comparison** | **Mode** | **m/z** | **RT** | **corr p-value** | **FC** |
| --- | --- | --- | --- | --- | --- | --- |
| **1,4-Cyclohexanedicarboxylic acid** | H_MCT vs C | RP neg | 171.0662 | 306.18 | 0.018 | 4.41 |
| **2-Aminoadipic acid** | BC_MCT vs C | HILC pos | 162.0762 | 764.83 | 0.025 | 0.68 |
|  | BC_MCT vs C | RP neg | 160.0615 | 56.27 | 0.037 | 0.57 |
| **2-Methylbutyrylcarnitine** | C_BC vs H | RP pos | 246.1701 | 293.95 | 0.014 | 0.36 |
| **3-Furoic acid** | H_MCT vs C | RP neg | 111.0089 | 98.88 | 0.036 | 2.35 |
| **Acetoacetic acid** | H_MCT vs C | RP neg | 101.0243 | 78.42 | 0.039 | 2.22 |
| **Acetylhistidine** | H_MCT vs C | HILIC neg | 196.0730 | 766.61 | 0.040 | 2.66 |
| **Acetyltaurine** | BC_ MCT vs C | RP pos | 168.0326 | 61.27 | 0.045 | 2.66 |
|  | BC_ MCT vs C | HILIC neg | 166.0180 | 365.71 | 0.027 | 2.13 |
| **Adipic acid** | BC_ MCT vs C | HILIC neg | 145.0506 | 127.93 | 0.039 | 6.00 |
|  | H_MCT vs C | HILIC neg | 145.0506 | 127.93 | 0.016 | 6.02 |
| **Alanine** | H_MCT vs C | HILC pos | 90.0549 | 720.29 | 0.037 | 2.44 |
| **Asparagine** | H_MCT vs C | HILIC neg | 131.0463 | 762.87 | 0.023 | 1.96 |
| **Citrulline** | H_MCT vs C | HILIC neg | 174.0885 | 806.20 | 0.038 | 2.03 |
|  | BC_ MCT vs C | HILC pos | 176.1027 | 797.03 | 0.020 | 2.30 |
|  | BC_ MCT vs C | RP neg | 174.0883 | 51.48 | 0.003 | 2.40 |
|  | H_MCT vs C | RP neg | 174.0883 | 51.48 | 0.019 | 1.73 |
|  | BC_ MCT vs C | RP pos | 176.1030 | 50.97 | 0.032 | 2.05 |
| **Dihydrouridine** | C_BC vs H | HILIC neg | 245.0780 | 196.00 | 0.008 | 0.63 |
|  | H_MCT vs C | HILIC neg | 245.0780 | 196.00 | 0.042 | 0.63 |
| **Dimethylglycin** | BC_ MCT vs C | HILC pos | 104.0705 | 684.78 | 0.012 | 2.34 |
| **Glutamine** | BC_ MCT vs C | HILIC neg | 145.0619 | 763.18 | 0.035 | 1.25 |
|  | H_MCT vs C | HILIC neg | 145.0619 | 763.18 | 0.040 | 1.72 |
|  | BC_ MCT vs C | RP neg | 145.0618 | 50.01 | 0.018 | 1.79 |
|  | H_MCT vs C | RP neg | 145.0618 | 50.01 | 0.007 | 1.82 |
| **Maleic acid** | C_BC vs H | RP neg | 115.0035 | 96.93 | 0.050 | 0.82 |
|  | H_MCT vs C | RP neg | 133.0142 | 66.73 | 0.042 | 2.86 |
| **Methionine** | H_MCT vs C | HILIC neg | 148.0439 | 610.48 | 0.039 | 2.13 |
|  | H_MCT vs C | RP pos | 150.0583 | 89.12 | 0.032 | 2.00 |
|  | H_MCT vs C | RP pos | 150.0583 | 89.12 | 0.032 | 2.00 |
| **N5-Acetylornithine** | BC_ MCT vs C | HILIC neg | 173.0932 | 764.16 | 0.029 | 4.56 |
|  | C_BC vs H | HILIC neg | 173.0932 | 764.16 | 0.024 | 0.34 |
| **N-Acetylglutamic acid** | H_MCT vs C | RP neg | 188.0564 | 115.84 | 0.048 | 2.13 |
| **N-Acetylglutamine** | H_MCT vs C | RP pos | 189.0869 | 76.12 | 0.045 | 3.02 |
|  | H_MCT vs C | HILIC neg | 187.0725 | 682.80 | 0.044 | 6.10 |
|  | BC_ MCT vs C | RP neg | 187.0724 | 77.93 | 0.037 | 3.13 |
|  | H_MCT vs C | RP neg | 187.0724 | 77.93 | 0.013 | 3.35 |
| **O-adipoylcarnitine** | H_MCT vs C | HILC pos | 290.1594 | 698.46 | 0.022 | 7.50 |
| **Pantothenic acid** | BC_ MCT vs C | HILC pos | 220.1177 | 201.64 | 0.039 | 1.92 |
|  | BC_ MCT vs C | RP pos | 220.1181 | 267.46 | 0.032 | 1.78 |
| **p-Hydroxyphenyllactate** | BC_ MCT vs C | HILIC neg | 181.0508 | 261.17 | 0.050 | 2.85 |
| **Proline** | BC_ MCT vs C | HILC pos | 116.0705 | 677.27 | 0.047 | 1.82 |
|  | H_MCT vs C | HILC pos | 116.0705 | 677.27 | 0.021 | 2.53 |
|  | H_MCT vs C | RP pos | 116.0706 | 54.98 | 0.015 | 2.16 |
| **Quinolinic acid** | H_MCT vs C | RP pos | 168.0291 | 87.76 | 0.029 | 7.57 |
| **Serine** | BC_ MCT vs C | HILIC neg | 104.0354 | 755.67 | 0.023 | 1.36 |
|  | BC_ MCT vs C | HILC pos | 106.0498 | 747.63 | 0.034 | 1.56 |
|  | BC_ MCT vs C | RP pos | 106.0499 | 48.85 | 0.032 | 1.44 |
| **Stearoylcarnitine** | BC_ MCT vs C | HILC pos | 428.3736 | 242.53 | 0.021 | 3.73 |
|  | H_MCT vs C | HILC pos | 428.3736 | 242.53 | 0.023 | 2.77 |
|  | BC_ MCT vs C | RP pos | 428.3735 | 519.52 | 0.001 | 5.99 |
|  | H_MCT vs C | RP pos | 428.3735 | 519.52 | 0.026 | 2.46 |
| **Suberic acid** | BC_ MCT vs C | HILIC neg | 173.0819 | 103.62 | 0.024 | 7.17 |
|  | H_MCT vs C | HILIC neg | 173.0819 | 103.62 | 0.033 | 10.43 |
| **Tetradecanoylcarnitine** | C_BC vs H | RP pos | 372.3108 | 468.15 | 0.045 | 0.40 |
| **Threonine** | H_MCT vs C | HILIC neg | 118.0510 | 727.49 | 0.042 | 1.74 |
| **Tyrosine** | H_MCT vs C | HILC pos | 182.0811 | 627.46 | 0.022 | 2.37 |
|  | H_MCT vs C | RP neg | 180.0666 | 126.66 | 0.007 | 2.15 |
|  | H_MCT vs C | RP pos | 182.0812 | 139.25 | 0.018 | 2.38 |
| **Uric acid** | BC_ MCT vs C | HILIC neg | 167.0211 | 398.05 | 0.035 | 1.55 |
|  | BC_ MCT vs C | RP neg | 167.0210 | 81.37 | 0.039 | 2.11 |

RT, retention time; FC, fold change; BC, breast cancer; H, healthy; MCT, long-chain triglyceride/ medium-chain triglyceride diet; C, control diet

**Supplementary Table S2.** Composition and energy supply of the different diets.

|  | CTRL | LCT/MCT8 |
| --- | --- | --- |
| Crude Protein % | 16.1 | 8.1 |
| LCT %^a^ | 7.1 | 49.6 |
| MCT8 %^b^ | 0 | 25 |
| MCT10 %^b^ | 0 | 0 |
| Sugar % | 6 | 1 |
| Starch % | 51.2 | 0 |
| Crude fiber % | 10 | 9.9 |
| Crude ash % | 4.5 | 4.4 |
| Energy Kcal | 3609 | 7098 |
|  | per Kg | per Kg |
| Vitamin A (IU/IE) | 15 | 15 |
| Vitamin D_3_ (IU/IE) | 1.5 | 1.5 |
| Vitamin E (mg) | 150 | 150 |
| Vitamin K_3_ (mg) | 20 | 20 |
| Vitamin C (mg) | 30 | 30 |
| Copper (mg) | 11 | 11 |

CTRL, control; MCT8, 8-carbon medium-chain triglyceride; ^a^ LCT composition: butter fat 11.7% and pork lard 88.3%; ^b^ MCT8 composition: pure oil

**Supplementary Table S3.** Settings of PeakPickerHiRes

| Parameter | Value |
| --- | --- |
| **PeakPickerHiRes** |  |
| version | 2.3.0 |
| processOption | inmemory |
| debug | 0 |
| threads | 1 |
| no_progress | false |
| force | false |
| test | false |
| **algorithm** |  |
| signal_to_noise | 0.1 |
| spacing_difference_gap | 4.0 |
| spacing_differnece | 1.5 |
| missing | 1 |
| ms_levels | 1 |
| report_FWHM | false |
| report_FWHM_unit | relative(ppm) |
| **SignalToNoise** |  |
| max_intensity | -1 |
| auto_max_stdev_factor | 3.0 |
| auto_max_percentile | 95 |
| auto_mode | 0 |
| win_len | 200.0 |
| bin_count | 30 |
| min_required_elements | 10 |
| noise_for_empty_window | 1.0E20 |
| write_log_messages | true |

**Supplementary Table S4.** Settings of FeatureFinderMetabo

| Parameter | Value |
| --- | --- |
| **FeatureFinderMetabo** |  |
| version | 2.3.0 |
| debug | 0 |
| threads | 1 |
| no_progress | false |
| force | false |
| test | false |
| **common** |  |
| noise_threshold_int | 3000.0 |
| chrom_peak_snr | 3.0 |
| chorm_fwhm | 5.0 |
| **mtd** |  |
| mass_error_ppm | 5.0 |
| reestimate_mt_sd | true |
| quant_method | area |
| trace_termination_criterion | outlier |
| trace_termination_outliers | 5 |
| min_sample_rate | 0.5 |
| min_trace_length | 0.25 |
| max_trace_length | -1.0 |
| **epd** |  |
| enabled | true |
| width_filtering | fixed |
| min_fwhm | 0.25 |
| max_fwhm | 60.0 |
| masstrace_snr_filtering | false |
| **ffm** |  |
| local_ret_range | 10.0 |
| local_mz_range | 6.5 |
| charge_lower_bound | 1 |
| charge_upper_bound | 3 |
| report_summed_ints | false |
| enable_rt_filtering | true |
| isotope_filtering_model | metabolites (5% RMS) |
| mz_scoring_13c | false |
| use_smoothed_intensities | true |
| report_convex_hulls | true |

**Supplementary Table S5.** Settings of FeatureLinkerUnlabeled QT

| Parameter | Value |
| --- | --- |
| **FeatureLinkerUnlabeledQT** | **Value** |
| version | 2.3.0 |
| keep_subelements | false |
| debug | 0 |
| threads | 1 |
| no_progress | true |
| force | false |
| test | false |
| **algorithm** |  |
| use_identifications | false |
| nr_partitions | 100 |
| ignore_charge | false |
| **distance_rt** |  |
| max_difference | 10.0 |
| exponent | 1.0 |
| weight | 1.0 |
| **distance_mz** |  |
| max_difference | 5.0 |
| unit | ppm |
| exponent | 2.0 |
| weight | 1.0 |
| **distance_intensity** |  |
| exponent | 1.0 |
| weight | 0.0 |

**Supplementary Table S6.** Settings of TextExporter

| Parameter | Value |
| --- | --- |
| **TextExporter** |  |
| version | 2.1.0 |
| replacement | _ |
| quoting | None |
| no_ids | False |
| debug | 0 |
| threads | 1 |
| no_progress | false |
| force | false |
| test | false |
| **feature** |  |
| minimal | false |
| add_metavalues | -1 |
| **id** |  |
| proteins_only | false |
| peptides_only | false |
| first_dim_rt | false |
| add_metavalues | -1 |
| add_hit_metavalues | -1 |
| **consensus** |  |
| sorting_method | None |
| sort_by_maps | false |
| sort_by_size | false |

**Supplementary Table S7.** Settings of R Snippets in KNIME workflow

| Parameter | Value |
| --- | --- |
| **Blank Filter_1** |  |
| blankFilterPassed | 14 |
| **ConsensusMapNormalization** |  |
| ignoreColsPattern | Blank |
| method | mean |
| outlier | 0.68, 1/0.68 |
| verbose | TRUE |
| **Pool Filter/RSD Filter** |  |
| poolFilterCount | 7 |
| maxRSD | 25 |
| **Biological Replicate Filter** |  |
| numInConditions | 3 |
| numInReplicates | 4 |

Supplementary Table S8. Settings of SIRIUS and CSI:FingerID

| Parameter | Value |
| --- | --- |
| Elements | CHNO P[5] S[8] Cl[1] |
| Compound timeout | 100 |
| Profile | Orbitrap |
| Filter by num masstraces | 2 |
| Feature only | true |
| Threads | 24 |
| All others | default |

**Supplementary Table S9.** Settings of METLIN database search

| Parameter | Value |
| --- | --- |
| Precursor Tolerance | 5 ppm |
| Collision Energy | 10/20/40 eV |
| MS/MS Tolerance | 0.01 Da |
| Mode | Positive/Negative |
| Adducts | all |
